# Wound Vitality in Decomposed Bodies: New Frontiers Through Immunohistochemistry

**DOI:** 10.3389/fmed.2021.802841

**Published:** 2021-12-24

**Authors:** Giuseppe Bertozzi, Michela Ferrara, Raffaele La Russa, Giovanni Pollice, Giovanni Gurgoglione, Paolo Frisoni, Letizia Alfieri, Stefania De Simone, Margherita Neri, Luigi Cipolloni

**Affiliations:** ^1^Department of Clinical and Experimental Medicine, Section of Legal Medicine, University of Foggia, Foggia, Italy; ^2^Department of Medical Sciences, Section of Legal Medicine, University of Ferrara, Ferrara, Italy

**Keywords:** wound vitality, decomposed body, IHC, GPA, MMP-9

## Abstract

**Background:** The question about wound vitality and the estimation of wound age of production are two of the classic investigation fields of forensic sciences. To answer this, the techniques most frequently used in research studies are immunohistochemistry (IHC), molecular biology, and biochemistry. Despite the great data on the literature about the usefulness of IHC in forensic pathology, there is always a request for further studies, especially on tissues altered by putrefactive phenomena. In fact, the degradation of the tissues is intended as the main limiting factor to the use of this technique.

**Scope:** The aim of this pilot study was to evaluate the immunohistochemical behavior of samples collected from decomposed bodies (in different putrefaction phases) and to relate these findings to wound vitality and postmortem interval.

**Materials and Methods:** Samples of skin and soft tissues were collected during autopsies, which were executed on decomposed bodies, whose cause of death was concluded to be traumatic. An immunohistochemical study was performed using antibodies against CD15, CD45, IL-15, tryptase, and glycophorin-A MMPs (endopeptidases involved in degrading extracellular matrix proteins: MMP-9 and MMP-2). An immunohistochemistry (IHC) reaction was evaluated according to a qualitative method as the following legend: (0): not expressed, (+): isolated and disseminated expression, (++): expression in groups or widespread foci, and (+++): widespread expression.

**Results:** Most of the tested markers (tryptase, glycophorin, IL15, CD 15, CD 45, and MMP9) showed to be highly expressed in the tissue of putrefied skin for 15 days.

**Discussion and Conclusion:** Although certainly inconclusive, this experimental application demonstrated that a nonexclusive but combined use of multiple antibodies is appropriate to verify wound vitality in decomposed bodies. Among them, GPA exhibited major reliability.

## Introduction

Assessment of the wound vitality is a long-standing question for forensic investigations to ascertain violent modality or supposed ones (e.g., in case of corpses found in open spaces, hypothetically wounded by local fauna after death) (1). To answer this, the most frequently used techniques in research studies are biochemistry molecular biology and immunohistochemistry (IHC) (2). Biochemical methods take advantage of the chemical and physics techniques. In particular, microspectrophotometry, microfluorimetry, and spectrophotometry have been used to assess concentration levels of vasoactive amines, although contradictory results emerged; also, atomic absorption spectrometry has been used to evaluate the diagnostic value of standalone ions and the ions ratio in skin wounds (3, 4).

Some authors found increased Fe concentrations in antemortem wounded skin and muscle, but no difference in Zn and Mg ions. Moreover, the K/Na ratio was found to be reduced in antemortem muscle samples, but not in the skin samples (5, 6).

On the other hand, molecular biology techniques have been applied rather onto the wound age estimation than on vitality (7–9). However, evaluation of mRNA levels of cytokines and enzymes throughout PCR technique has its rationale in the occurring changes of mRNA levels, after wounding, sooner than protein levels and histomorphology alterations (1, 10, 11). Nevertheless, the degradation of RNA caused by post-mortem effects is the most probable occurrence in some days. Hence, by measuring specific mRNA levels into the known decay time, it is possible to estimate the wound age estimation.

However, among all the techniques, IHC provides a great deal of evidence in the literature, demonstrating to be a valuable choice in determining, with a wide variety of markers (tissue molecules, cytokines, and growth factors), if a lesion is vital or not (2, 12). Furthermore, the IHC, if compared to other techniques, has proved to be more useful not only for its ease of application and its high reliability but, above all, for the possibility to analyze the localization of the molecules of interest (13). In this context, even if some markers are promising, prior to their application in daily routine, their use needs to be confirmed with other studies.

However, although these techniques are continuously studied in the forensic field of vitality on samples collected from fresh cadavers, there are not many applications on the decomposed bodies due to degeneration of microstructures investigated through routinely accessible methods. In particular, the skin samples are harder to be studied because of the ease of putrefaction in comparison to the muscle tissues located in the interior of the body (14). Indeed, to our knowledge, no biochemical method-based study has been performed extensively on putrefied specimens, as well as there are no molecular biology universal applications. Even with the IHC efficacy, wound vitality evaluation in putrefied corpses varies among authors in literature, mainly depending on each marker sensibility and specificity; moreover, the leakage of searched antigens normally contained into known structures produces a lack of specificity (15).

In this context, the aim of this pilot study was to evaluate the immunohistochemical behavior of samples collected from the decomposed bodies (in different putrefaction phases) and to relate these findings to wound vitality and post-mortem interval (PMI).

## Materials and Methods

### Case Selection

The cases for the present study were selected as follows: (i) cadavers with residual skin; (ii) cadavers not subjected to special transformative processes (such as saponification and/ or corification); and (iii) cadavers whose evidence gathered from the circumstantial data, crime scene investigation, external inspection until the mascroscopic and microscopic autoptic findings, oriented toward a traumatic death. Therefore, a total of 7 case studies, with different stages of decomposition between a few hours and 15 days after death, were elected. A negative control (NC) case (a decomposed body with no-traumatic injuries but with skin losses thorough feeding activity of the local macro- and microfauna) was also included. The result of the selection is summarized in Table 1.

**Table 1:** Cases and control characteristics.

**Case**	**Historical** **data**	**Intrinsic features**	**Environmental condition**	**PMI**	**Cause of death attributed**	**Sample**
CASE 1	Suicide	Nomal adipose panniculus; dressed	Housing; door and windows closed	24–36 h from death	Gunshot	Lacerated wound of the frontal region
CASE 2	Fight among partners	Normal adipose panniculus; dressed	Housing; door and windows closed	3–6 days from death	Strangulation	Anterior region of the neck
CASE 3	suicide	Nomal adipose panniculus; dressed	Countryside, outdoors; dry and ventilated climate	3–6 days from death	Hanging	Anterior region of the neck
CASE 4	Family mourning	Aboundant adipose panniculus; dressed	Countryside, outdoors; humid and unventilated climate	7–10 days from death	Slaughtering	Incised wound of the neck
CASE 5	Agricultural worker	Normal adipose panniculus; dressed	Countryside, outdoors; dry and ventilated climate	7–10 days from death	Massive fracture head injury	Lacerated wound of the scalp
CASE 6	Frequent allegations of assault	Normal adipose panniculus; dressed	Countryside, outdoors; dry and unventilated climate	10–15 days from death	Burst head injury	Lacerated wound of the scalp
CASE 7	Disappearance by family members reported	Aboundant adipose panniculus; dressed	Mountain forest, outdoors; dry and ventilated climate	10–15 days from death	Slaughtering	Incised wound of the neck
CASE 8 (NC)	Disappearance by family members reported	Aboundant adipose panniculus; dressed	Countryside, outdoors; dry and ventilated climate	3–6 days from death	Sudden cardiac death	Skin losses by macro- and micro-fauna from the arms

### IHC

All the samples of tissue have been fixed in formalin 10% for 48 h, then processed and included in paraffin. For each sample, 4 micron thick sections have been carried out; one section has been stained with Haematoxylin and Eosin (H&E). On the other sections, an immunohistochemical study has been performed using a panel of antibodies; details as summarized in the following Table 2.

**Table 2:** A panel of antibodies in study.

**Tryptase**			
(mouse monoclonal antibody, sc-59587, Santa Cruz, CA, USA)	Proteinase K (T: 20 °C for 15 min)	120 min, 20°C	1:1000
IL-15 (mouse monoclonal antibody, sc-8437, Santa Cruz, CA, USA)	boiling in 0.25 mM EDTA buffer.	120 min, 20°C	1:50
CD 15 (mouse monoclonal antibody, sc-53290, Santa Cruz, CA, USA)	boiling in 0.25 mM EDTA buffer.	120 min, 20°C	1:50
MMP 2 (mouse monoclonal antibody, sc-53630, Santa Cruz, CA, USA)	boiling in 0.1 M Citric Acid buffer.	120 min, 20°C	1:100
MMP 9 (mouse monoclonal antibody, sc-21733,Santa Cruz, CA, USA)	boiling in 0.1 M Citric Acid buffer.	120 min, 20°C	1:100
Glycophorin A (GPA - mouse monoclonal antibody, sc-59181, Santa Cruz, CA, USA)	boiling in 0.25 mM EDTA buffer	120 min, 20°C	1:500
CD 45 (mouse monoclonal antibody, sc-19664, Santa Cruz, CA, USA)	boiling in 0.25 mM EDTA buffer.	120 min, 20°C	1:600

The sections in paraffin have been rehydrated and incubated for 20 min in methanol, containing 10% of H_2_O_2_ to block endogenous peroxidases. The sections have been pre-treated to facilitate antigen retrieval and to increase membrane permeability to antibodies, then incubated with the primary antibody (Table 1). The utilized detection system was a refined avidin–biotin system in which a biotinylated secondary antibody reacts with several peroxidise-conjugated streptavidin molecules. The positive reaction was visualized by 3,3′-diaminobenzidine (DAB) peroxidation, according to standard methods. The sections were counter-stained with Mayer's hematoxylin, dehydrated, cover-slipped, and observed with an optical microscope.

Histologic examination was based on a semiquantitative screening, of which we report below the gradation of positive reaction:

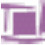
 [0]: not expressed,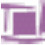 (+): isolated and disseminated expression,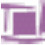 (++): expression in groups or widespread foci,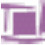 (+++): widespread expression.

## Results

**Figure 1:**
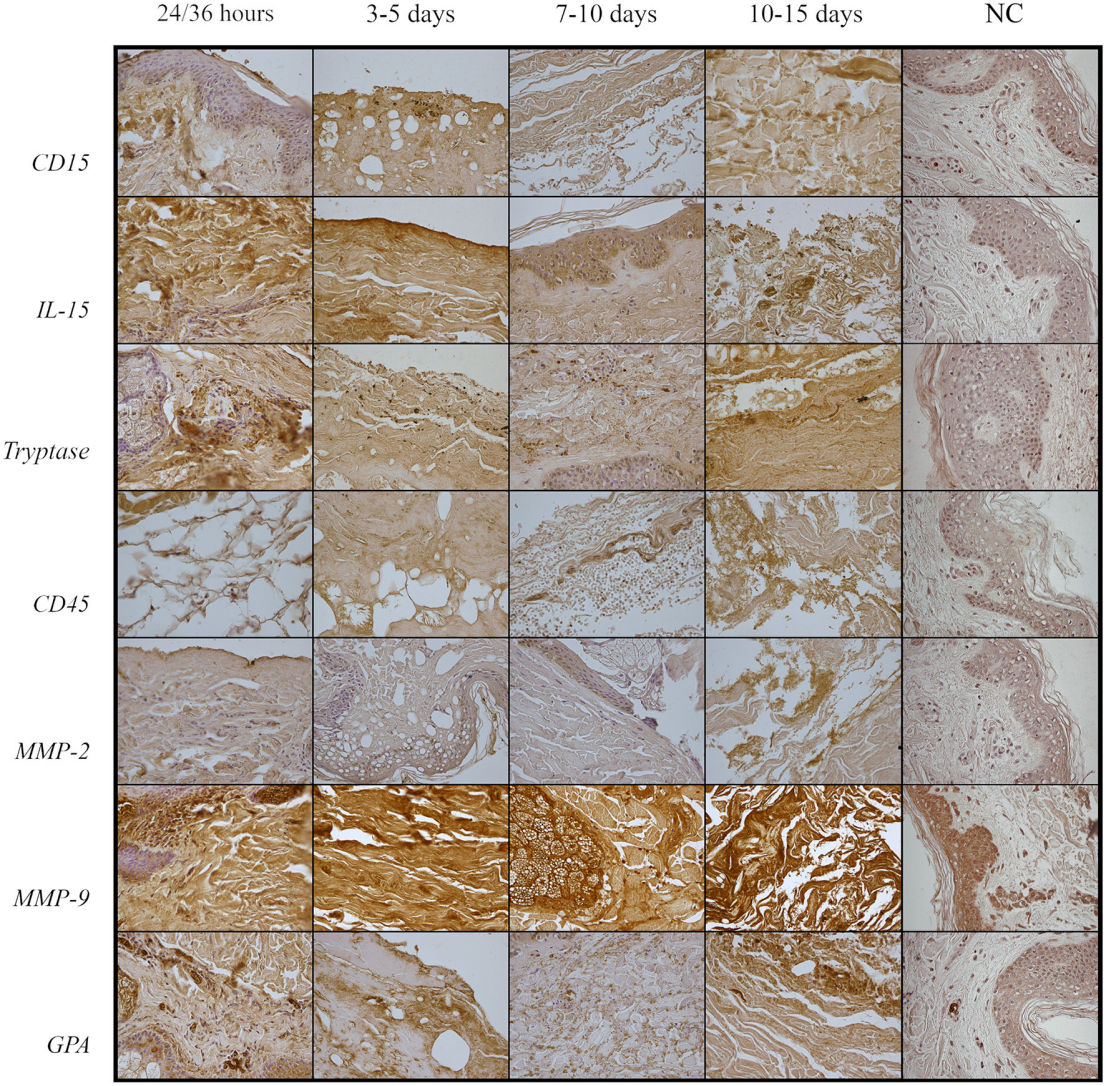
Differences in IHC reactions according to PMI.

The microscopic analysis of the skins' preparations showed a different reactivity in various stages of putrefaction. Among the different tested antibodies, only some showed a remarkable reactivity on the very particular putrefied tissue of skin (Table 2). Based on this first observation, we selected some markers, which were expressed in the putrefied tissues. Most of the tested markers (tryptase, GPA, IL15, CD 15, CD 45, and MMP9) were shown to be highly expressed in the tissue of putrefied skin for up to 15 days. The reaction against antibody anti-CD 15, CD45, and GPA is localized on the cellular membrane; tryptase, IL15 MMP2, and MMP9 antibodies showed cytoplasmic staining. All measurements were done on the same magnification of image (40 x) and by three different examiners (Table 3). A correlation between the IHC pattern and the PMI is also provided in Figure 1.

**Table 3:** IHC reaction evaluation according to the qualitative method selected by three different examiners.

**Antibody**	**CASE 1**	**CASE 2**	**CASE 3**	**CASE 4**	**CASE 5**	**CASE 6**	**CASE 7**	**CASE 8**
CD 15	+++	+++	+++	++	++	++	++	-
IL-15	+++	+++	+++	++	++	++	++	-
Tryptase	+++	+++	+++	++	++	++	++	-
CD 45	+++	+++	+++	++	++	+	+	-
MMP 2	++	+	+	+	+	+	+	-
MMP 9	+++	+++	+++	+++	+++	+++	+++	+
GPA	+++	+++	+++	+++	+++	+++	+++	-

## Discussion

Diagnosis of wound vitality and wound-age estimation represent crucial, and, still open, questions for forensic pathologists. Vitality refers to a set of physiological processes, such as erythrocyte extravasation or inflammation, which prove the injury was inflicted when the individual was still alive. Typically, the vitality of a lesion was assessed with the standard hematoxylin-eosin stain to detect erythrocyte extravasation. However, this evidence alone does not represent a reliable sign of vitality, as some studies have suggested that red blood cells extravasations can occur even after death (16, 17).

Wound-age estimation remains an unsolved problem that is limited by the non-specificity and low reproducibility of biomarkers, as well as the ethical limitations related to the impossibility of excising wound samples in living subjects (1).

The numerous studies published in the literature have identified immunohistochemistry, molecular biology, and biochemical tests as the tools to solve these questions. Immunohistochemistry is the most widely used method due to its ability to detect the location of antigens, ease application on formalin-fixed paraffin-embedded tissue, and reproducibility (1).

The forensic science community has focused its attention on various immunohistochemical markers to differentiate pre- and post-mortem injuries and to estimate the time interval between the infliction of the wound and the death. Among the investigated molecules are inflammatory cytokines, coagulation factors, metal ions, structural proteins of erythrocytes, and proteolytic enzymes that were involved in the physiological response of a living tissue to external stimuli (6, 18–20). Immunohistochemical methods gave satisfying results in recently deceased bodies; however, their use is challenging in putrefied corpses, as the alteration of the tissues can compromise the interpretation of the results. The main limitation linked to putrefaction is the degradation of the protein molecules, which alters the antigens and determines their migration to a different site than the original one. In addition, a false positivity may arise in putrefied tissues due to the increased binding of antibodies to the altered epitopes. Nevertheless, several studies have tested the applicability of immunohistochemistry on decomposed corpses in different tissues and have found that some antigens can be identified despite the tissue alteration. The most significant applicability of immunohistochemistry was observed within a post-mortem interval of 3 days (21).

Glycophorin A (GPA), an integral membrane protein of erythrocytes, is a widely used bleeding marker. In putrefied subjects, it can highlight bleeding in the skin, bone, and muscle tissue. According to Tabata and Morita, immunohistochemical detection of GPA in the putrefied corpse allows discrimination between hemoglobin diffusion and bleeding (1, 22). The present study has tested the possibility of applying immunohistochemistry on skin samples taken from traumatic lesions of corpses in different stages of putrefaction, using a panel of markers involved in the mechanisms of inflammation and wound repair: metalloprotease 2 and 9 (MMP-2 and MMP-9), interleukin 15 (IL-15), tryptase, and leukocyte differentiation clusters 15 and 45 (CD15 and CD45); in addition, GPA was used to identify bleedings.

MMPs are proteolytic enzymes expressed proactively in tissues, which are converted into active form after injury. They take part in a variety of processes, including remodeling of extracellular matrix and cell repair. The MMP-2 and MMP-9 are implicated in collagen, elastin, fibronectin, and laminin degradation, and have pro- and anti-inflammatory roles in numerous tissues (23). The MMPs are proteolytic enzymes proactively expressed in tissues, which are converted into active form after injury. In particular, MMP-2 and MMP-9, respectively known as gelatinases A and B, take part in a variety of processes, including: (i) remodeling of extracellular matrix and cells repair; (ii) degradation of collagen, elastin, fibronectin, and laminin; (iii) digesting various inflammation-involved molecules, such as pro-TNF, TGF-β, pro-IL-1β, and pro-IL-8; and (iv) processing of various pro- and antiangiogenic factors during wound healing (24, 25). Although they are not reliable in the diagnosis of viability as they do not differ significantly in pre- and post-mortem lesions, several studies emphasize their usefulness in determining the wound age (26). Indeed, increased expression of MMP-2 can be detected in the connective tissue at the edge of acute wounds during all stages of the healing process and remained fairly stable until the phase of re-epithelialization. The MMP-9 contributes to healing wound by the initiation of keratinocytes migration and mobilization of endothelial progenitor cells from the bone marrow (27, 28).

The present study findings show that MMP-2 is widely expressed in the recently deceased corpse, while it decreases between 3 and 6 days, respectively, after death, up to be weak/absent in the 10–15 days post-mortem interval. Conversely, MMP-9 expression is unvaried in the different putrefaction phases.

The IL-15 is a cytokine with a wide range of functions. It is involved in the recruitment of mononuclear cells, deposition of fibrous tissue, angiogenesis, and regulation of the phenotype of lymphocytes and monocytes. Together with CD-15 (an antigen expressed by leukocytes, involved in cell adhesion), IL-15 appears to be a reliable indicator for assessing the viability of lesions (29), although there are no studies on its applicability in putrefied bodies (30).

Gauchotte et al. indicated that CD-15 positive cells can be passively released from the vessels in putrefied samples, thus giving false positives (15). This research results highlight a widespread expression of IL-15 and CD15 of up to 6 days after death, which decreases with the advancement of putrefactive phenomena.

The CD45 is a leukocyte antigen used to assess the viability of the lesion, as its immunohistochemical detection localizes migrated white blood cells to the site of inflammation. Furthermore, it is a reliable marker for estimating the time of the injury (31). The present results demonstrate strong CD45 expression within a 6-day post-mortem interval, which progressively decreases over time.

Tryptase is a protease contained in mast cell granules, involved in the inflammatory and anaphylactic response. Its immunohistochemical identification suggests the activation and consequent degranulation of mast cells, and, therefore, is an indicator of the vitality of the lesion (32). According to this study, tryptase is strongly expressed within 6 days of death and weakens as the post-mortem interval increases. Furthermore, the expression of GPA remains constant despite the time progresses.

However, this study does not lack limitations, represented, above all, by the small number of samples included and by the non-uniformity of the environmental conditions at the time of the discovery of the corpses. Moreover, the present work has focused its attention on a post-mortem interval of 15 days; thus, the results do not allow for considerations beyond this time.

## Conclusion

The present study demonstrates the possibility of investigating wound vitality through the IHC method, despite the limitations inherent to the technique itself and relative to its application in samples from decomposed bodies. Indeed, the positivity of the selected antibodies has been noticed for up to 15 days from death, except for MMP-2, which was definitely positive in the only corpse with the most recent PMI. Furthermore, the positivity obtained turns out to be inversely proportional to the PMI. Therefore, to obtain a diagnosis of vitality as accurate and reliable as possible, a non-exclusive but combined use of these markers is recommended in decomposed bodies. On the other hand, when selecting the other antibodies to be used, it is necessary to keep in mind the non-unique interpretation of the data provided by the literature review, particularly regarding the use of MMP-9. Besides, the CD15-positive cells could passively disseminate from the vessels altered by the decomposition process. However, in this integration of evidence, the antibody that was proven to be more reliable, and should be routinely used is GPA. Further studies are, therefore, needed to standardize the antibody pattern to be used.

## Data Availability Statement

The raw data supporting the conclusions of this article will be made available by the authors, without undue reservation.

## Author Contributions

LC and GB: conceptualization and supervision. GB and MN: methodology. MF and RLR: validation. Autoptic cases from SDS, LC, MN. MF, GG, and GP: literature review. PF, LA, and MN: IHC investigation. GB, MF, GG, and GP: writing of original draft preparation. RLR, PF, LC, and MN: writing, reviewing, and editing. All authors have read and agreed to the published version of the manuscript.

## Conflict of Interest

The authors declare that the research was conducted in the absence of any commercial or financial relationships that could be construed as a potential conflict of interest.

## Publisher's Note

All claims expressed in this article are solely those of the authors and do not necessarily represent those of their affiliated organizations, or those of the publisher, the editors and the reviewers. Any product that may be evaluated in this article, or claim that may be made by its manufacturer, is not guaranteed or endorsed by the publisher.
